# Nutraceuticals in Thyroidology: A Review of in Vitro, and in Vivo Animal Studies

**DOI:** 10.3390/nu12051337

**Published:** 2020-05-08

**Authors:** Salvatore Benvenga, Silvia Martina Ferrari, Giusy Elia, Francesca Ragusa, Armando Patrizio, Sabrina Rosaria Paparo, Stefania Camastra, Daniela Bonofiglio, Alessandro Antonelli, Poupak Fallahi

**Affiliations:** 1 Master Program on Childhood, Adolescent and Women’s Endocrine Health, Department of Clinical and Experimental Medicine, University of Messina, 98125 Messina; s.benvenga@live.it; 2 Interdepartmental Program of Molecular & Clinical Endocrinology, and Women’s Endocrine Health, University Hospital, Policlinico Universitario G. Martino, 98125 Messina, Italy; 3 Department of Clinical and Experimental Medicine, University of Pisa, 56126 Pisa, Italy; sm.ferrari@int.med.unipi.it (S.M.F.); e.giusy_87@hotmail.it (G.E.); francescaragusa86@gmail.com (F.R.); armandopatrizio125@gmail.com (A.P.); sabrinapaparo@gmail.com (S.R.P.); stefania.camastra@unipi.it (S.C.); 4 Department of Pharmacy, Health and Nutritional Sciences, University of Calabria, 87036 Arcavacata di Rende (CS), Italy; daniela.bonofiglio@unical.it; 5 Department of Translational Research and New Technologies in Medicine and Surgery, University of Pisa, 56126 Pisa, Italy; poupak.fallahi@unipi.it

**Keywords:** nutraceuticals, thyroid, carnitine, flavonoids, melatonin, omega-3, resveratrol, selenium, vitamins, zinc

## Abstract

Nutraceuticals are defined as a food, or parts of a food, that provide medical or health benefits, including the prevention of different pathological conditions, and thyroid diseases, or the treatment of them. Nutraceuticals have a place in complementary medicines, being positioned in an area among food, food supplements, and pharmaceuticals. The market of certain nutraceuticals such as thyroid supplements has been growing in the last years. In addition, iodine is a fundamental micronutrient for thyroid function, but also other dietary components can have a key role in clinical thyroidology. Here, we have summarized the in vitro, and in vivo animal studies present in literature, focusing on the commonest nutraceuticals generally encountered in the clinical practice (such as carnitine, flavonoids, melatonin, omega-3, resveratrol, selenium, vitamins, zinc, and inositol), highlighting conflicting results. These experimental studies are expected to improve clinicians’ knowledge about the main supplements being used, in order to clarify the potential risks or side effects and support patients in their use.

## 1. Introduction

The term “nutraceutical” is placed in an area among food, food supplements, and pharmaceuticals [1]. Nutraceuticals are considered complementary medicines, defined as a “food, or parts of a food, that provide medical or health benefits, including the prevention and treatment of disease” [2]. Most nutraceuticals are normal human metabolites (i.e., dehydroepiandrosterone (DHEA) and S-adenoylmethionine (SAMe), carnitine, creatine, coenzyme Q10, lipoic acid, melatonin), or bioactive plant dietary components [2]. Food categories and supplements are both described in the European regulation (No. 1924/2006 of the European Parliament and of the Council, updated by EU Regulation 2015/2283), even if an official mention or recognition of the term “nutraceutical” does not exist [3]. The European Food Safety Authority (EFSA) does not distinguish clearly the terms “food supplements” and “nutraceuticals, while in America “medical foods” and “dietary supplements” are regulatory terms, although “nutraceuticals” and “functional foods” are determined according to consumer trends [1].

In the last years, interest and knowledge in nutraceuticals have been growing. Nutraceuticals can be considered for the prevention of different pathological conditions, including thyroid diseases and associated disorders [4,5,6]. 

In addition to iodine, which is a fundamental nutrient for thyroid function, other dietary components (such as carnitine, flavonoids, melatonin, omega-3, resveratrol, selenium, vitamins, zinc, and inositol) were found to have some role in thyroid homeostasis, so that they could have a role in clinical thyroidology. The principal issue about the appropriateness and effectiveness of nutraceuticals in prevention and treatment depends on the lack or scarcity of clinical data [1]. Moreover, there is the problem of the not uncommon discrepancy between the concentration reported in the label and the real one [1]. Conventional medicines are usually submitted to quality control to ensure that they contain the claimed dose of active constituents, and that they have suitable disintegration characteristics and bioavailability, enabling absorption in the gut tract. Composition of nutraceuticals is increasingly being evaluated, the results of the analyses being that composition sometimes fails the relevant standards, or the label claims are not respected [2].

Here, we aim to review various nutraceuticals that can influence human thyroid homeostasis, addressing on the in vitro, and in vivo experimental animal studies reported in literature. We will focus on nutraceuticals, other than iodine, that are more likely to be encountered in the clinical practice. 

## 2. Search of the Literature

A PubMed search, run on March 2020, using the word “nutraceuticals” as the entry, yielded 74,935 results, indicating the great interest in general for this emerging class of natural compounds that makes the line between food and drugs to fade. Interestingly, using the entry “nutraceuticals AND thyroid” a total of 6622 published papers were obtained, highlighting that the scientific interest of nutraceutical area covers the thyroid research field. Indeed, different nutraceuticals possibly influence human thyroid function and/or thyroid tumor biology that will be reviewed and commented upon. Particularly, using the filter “humans” to exclude “other animals”, and the filter “other animals” in order to exclude “humans”, we have meticulously screened the in vitro and in vivo experimental studies on thyroid and carnitine (“carnitine AND thyroid”), flavonoids (“flavonoids AND thyroid”), isoflavonoids (“isoflavonoids AND thyroid”), soy (“soy AND thyroid”), melatonin (“melatonin AND thyroid”), omega-3 polyunsaturated fatty acids (“omega-3 polyunsaturated fatty acids AND thyroid”), resveratrol (“resveratrol AND thyroid”), selenium (“selenium AND thyroid”), vitamins (“vitamins AND thyroid”), zinc (“zinc AND thyroid”), and inositol (“inositol AND thyroid”) (Table 1).

## 3. Carnitine

The naturally occurring quaternary amine, carnitine, is ubiquitous in mammalian tissues, and according to studies of about 40 years ago, it was considered a peripheral antagonist of thyroid hormone (TH) action [7].

Old studies published in German language showed that carnitine is capable of contrasting TH-induced changes associated with the nitrogen balance in rats and metamorphosis of tadpoles [8,9]. In the more recent of such papers, carnitine contrasted the thyroxine (T4)-induced liver and circulating concentration of both alanine aminotransferase (ALT) and aspartate aminotransferase (AST) [9]. Tissue culture experiments on human skin fibroblasts, human hepatoma cells HepG2, and mouse neuroblastoma cells NB 41A3 demonstrated that L-carnitine inhibits cell entry, and overall nuclear entry, of triiodothyronine (T3) and T4 [7]. There was no inhibition on TH efflux from cells, and no inhibition of TH binding to isolated nuclei. These data confirm that carnitine is a peripheral antagonist of TH action, and that one level of inhibition occurs at the nuclear envelope or before it [7].

Four experimental groups were formed starting from 21 male Sprague Dawley rats: hyperthyroidism (*n* = 5), hyperthyroidism plus low dose L-carnitine (100 mg/kg/d for 10 days; *n* = 5), hyperthyroidism plus high dose L-carnitine (500 mg/Kg/d for 10 days; *n* = 5), and controls (0.2 mL/100 g body weight, subcutaneously, of 0.9% NaCl solution; *n* = 6) [10]. The injection of levothyroxine (L-T4) in a dose of 250 μg/kg body weight per day for 20 consecutive days was able to induce hyperthyroidism in rats. The treatment with either dose of L-carnitine was by intraperitoneal injection, and it started on the 10th day of hyperthyroidism continuing for the following 10 days. Activities of one marker of oxidative stress (malondialdehyde (MDA)) and activities of three markers of antioxidant defense (namely, the antioxidant enzymes catalase (CAT), glutathione peroxidase (GPX), and myeloperoxidase (MPO)) were measured in liver homogenates. MDA activity was increased by 59% in the carnitine-untreated hyperthyroid group, but it decreased significantly and to levels comparable to the control rats in either group of hyperthyroid rats receiving L-carnitine. Activities of the three enzymes were 21% to 76% lower in the carnitine-untreated hyperthyroid rats with respect to the control group. Treatment of hyperthyroid rats with either low or high dose of L-carnitine increased strongly the liver activities of the antioxidant enzymes (with dose-dependency absent for CAT, moderate for GPX and great for MPO), indicating that even a low dose of L-carnitine was enough to prevent the oxidative stress induced in the rat liver by L-T4 [10].

Some experimental data are available in the neoplastic setting of the thyroid. L-carnitine, the biologically active form of carnitine, transports long-chain acyl groups from fatty acids into the mitochondrial matrix to generate metabolic energy in living cells. Although, it has been reported that treatment with L-carnitine efficiently induced ATP generation in normal cells, it has been found to selectively inhibit cancer cell growth in vitro and in vivo models [11]. 

Controversially, the expression of the enzyme involved in this transport, the carnitine palmitoyltransferase 1C (Cpt1c), has been detected at higher levels in papillary thyroid tissues compared with normal ones and Cpt1c up-regulation has been found to promote cancer cell growth and metastasis in human papillary thyroid carcinomas cell lines [12].

Recently, carnitine has been reported as a potential candidate biomarker able to discriminate between normal and thyroid cancer cells, however, further studies are needed to confirm carnitine as the thyroid cancer diagnostic oncometabolite [13]. Of interest, a recent Turkish study [14] used 40 guinea pigs to assess the protective effects of amifostine (200 mg/kg ip), L-carnitine (200 mg/kg ip), or vitamin E (40 mg/kg im) against high dose radioactive iodine (131I) treatment-induced salivary gland damage. Control animals received ^131^I was administered intraperitoneally at doses (555–660 MBq) that ablate the thyroid and impair the parenchymal function of the salivary glands. The damage of the salivary glands was evaluated one month after treatment, by salivary gland scintigraphy and histopathology, in 40 guinea pigs. The three molecules gave different levels of protection against radioactive iodine treatment injury in salivary glands; however, none of the agents could provide absolute protection.

## 4. Flavonoids, Isoflavonoids, Soy

Flavonoids are the most common group of polyphenolic compounds in the human diet and are widespread in plants [15], and they can be classified into flavonoids or bioflavonoids; isoflavonoids; and neoflavonoids. It has been reported that flavonoids can interfere with thyroperoxidase (TPO) activity, reducing TH synthesis with subsequent raise of thyroid-stimulating hormone (TSH) levels and potential development of goiter. Goiter occurrence has been described among infants fed with soy formula, while the thyroid profile was normal in post-menopausal women with regular soy diet. Moreover, flavonoids seem to impair the peripheral action of TH, by the inhibition of deiodinase or displacing T4 from transthyretin [16]. Recently, the debate on soy foods and diet has earned attention among the healthcare and general public. 

Since isoflavones from soy and other legumes showed to act on estrogen pathway, they are also proposed as nutraceutical products to relieve women from symptoms of menopause [17]. However, data regarding the impact of isoflavones on endogenous estrogens levels in women are still controversial. To date, no health issue on isoflavones has been ratified by EFSA because of insufficient scientific evidence, while the available human studies ruled out the hypothesis of adverse effects of isolated isoflavones on mammary gland, uterus or thyroid health among postmenopausal women. Nevertheless, there are many divergences to consider in term of metabolism of isoflavones, developmental stage at time of consumption and in their temporarily restricted uptake during certain stages of life, that make animal models not reliable for humans. Thus, potential adverse effects cannot be completely ignored, especially among women with unknown diseases status (i.e., undetected precancerous lesions in the mammary gland) [17].

In 2014, a review explored 5 health benefits-relieves of menopausal symptoms and prevention of breast cancer, heart disease, osteoporosis, and prostate cancer, and 5 health risks-increased risk of breast cancer, hypothyroidism, male hormonal and fertility problems, antinutrient content, and harmful processing by-products [18]. The authors considered in their analysis prospective human trials, systematic reviews of human trials, observational human studies, in vitro studies, laboratory analyses of soy components, and animal studies. They noticed that isoflavones and soy foods may wane menopausal symptoms and protect from breast cancer and heart disease, but not from osteoporosis. The impact on male fertility and reproduction was controversial. With regard to thyroid activity, data are conflicting and there is uncertainty, demonstrating that soy may have unpredictable effect on thyroid physiology [18].

In a study, adult female cynomolgus monkeys (Macaca fascicularis) were randomized in 2 groups, according to diet: One to consume casein-lactalbumin (*n* = 44) and the other soy protein with isoflavones (*n* = 41) [19]. All animals were ovariectomized after 34 months, and then, for other 34 months, half of the monkeys from each diet treatment group continued to receive their preovariectomy diet. The remaining animals were not considered furtherly. The authors concluded that soy protein and isoflavones do not adversely affect thyroid function in females [19].

Anyway, studies of soy isoflavones in experimental animals suggest possible adverse effects as well (i.e., anti-thyroid effects, modulation of endocrine function, and enhancement of reproductive organ cancer) [20]. 

A study showed that rats fed with a diet containing soy (20% defatted soy bean) had a severe hypothyroid state (low T4, increased TSH and thyroid weight), with evidence for increased thyroid cell proliferation. This hypothyroidism was induced only when a dietary condition of iodine deficiency was added [21].

Another paper indicated a dramatic synergism between soy intake and iodine deficiency on the induction of thyroid hyperplasia in rats [22]. Female F344 rats were randomized into 8 groups, and for a 5-week period received a diet containing: 1) 0.2% soy isoflavone mixture (SI); 2) 0.2% SI + iodine deficiency (ID); 3) 0.04% SI; 4) 0.04% SI + ID; 5) 20% defatted soybean (DS) alone; 6) 20% DS + ID; 7) ID alone; 8) basal diet alone. In the group receiving 20% DS, serum T4 and TSH levels increased inducing thyroid growth in rats exposed also to the ID diet. In the ID diet groups, proliferating cell nuclear antigen labeling indices (%) were elevated and increased by DS, but not SI, suggesting that isoflavones may not participate in the mechanisms underlying the synergistic goitrogenic effect of soybean with iodine deficiency [22].

Genistein (4′,5,7-trihydroxyflavone) is a phytoestrogen that belongs to the class of soy isoflavones and is effective to treat osteoporosis, menopausal vasomotor symptoms, cardiovascular diseases, as well as a variety of cancers. Little is known about the action of isoflavones on thyroid integrity in humans, even if it seems that genistein does not act negatively on thyroid safety in euthyroid humans [23]. Recently, it has been demonstrated that genistein has antineoplastic effects, but it does not induce genotoxic effects whereas it decreases oxidative-induced DNA damage in human primary thyroid cells from papillary thyroid cancer, supporting its potential use in therapeutic intervention [24].

A study evaluated the biological effects of genistein in rats receiving genistein aglycone in soy-free feed fortified at 0, 5, 100, and 500 ppm, beginning in utero through 20 weeks [24]. In rat serum, the genistein content was of 8 μM, and it increases in thyroid tissues up to 1 pmol/mg both in male and female rats. The activity of TPO was reduced by up to 80% dose-dependently in rats of both gender. Male and female rats receiving a standard soy-based rodent diet had TPO activity ~50% lower than rats consuming a soy-free diet. Comparing treated and untreated groups, there were no differences in T3, T4, and TSH serum levels, thyroid weights, and histopathology. The reported data suggested that, even if normal rats lose partial activity of TPO when they receive soy isoflavone, thyroid homeostasis is guaranteed by remaining enzymatic activity [25].

Quercetin is the most abundant dietary flavonoid in fruit and vegetables, and it has different therapeutic actions, i.e., the induction of apoptosis in cancer cells, and antioxidant, antiviral, anti-proliferative, and anti-inflammatory effects [26]. Regarding the thyroid, many studies have shown anti-thyroid and goitrogenic effects of flavonoids, different according to each specific flavonoid [16].

As a pretreatment for Wistar rats, quercetin was administered orally at the dose of 10 mg/kg for 7 days, and it protected them from myocardial infarction induced by subcutaneous injection of isoproterenol. The ST-segment elevation was lowered and levels of lipid peroxidation products were decreased in plasma and heart [27]. Moreover, the pretreatment with quercetin reduced significantly the levels of total cholesterol, triglycerides and free fatty acids in serum, heart, and heart mitochondria and serum phospholipids, and it lowered levels of serum LDL and very LDL cholesterol, while raised significantly serum HDL [27]. 

When quercetin was given (0.1%; w/w in diet) to human CRP transgenic mice, a humanized inflammation model, and ApoE*3Leiden transgenic mice, a humanized atherosclerosis model, it halted IL-1b-induced CRP expression in the first and lowered the burden of atherosclerosis (40%) in the second through a reduction of circulating inflammatory markers, “serum amyloid A proteins” and fibrinogen. The quercetin plasma levels (13–19 mM) were similar among both groups and to those measured in rodents treated with the same doses (0.1%, w/w) [28].

In 2008, quercetin was shown to halt the spread in FRTL-5 thyroid cells dose- and time-dependently, by inhibiting insulin-regulated Akt kinase action [29]. Quercetin interferes with TSH-dependent NIS gene expression and I- transport in FRTL-5 cells. These observations may help us to understand the molecular mechanism of the antithyroid effect of quercetin on cell growth and function. Even if collected from an in vitro, hormonally controlled, functioning thyroid cell line, that does not have the characteristics of a transformed cell, these results led to evaluate quercetin as an antithyroid drug in hyperfunctioning states [29].

In recent studies, quercetin seems to reduce the expression of the thyrotropin receptor, TPO and thyroglobulin (Tg) genes [30]. The antithyroid impact of quercetin was further evaluated in vivo: Quercetin was administered (50 mg/kg) to a Sprague–Dawley rat and after 14 days of treatment, radioiodine uptake decreased significantly demonstrating that quercetin may act as a thyroid disruptor [30].

Apigenin, a plant-derived flavonoid, has been also considered able to increase the iodide influx through Akt inhibition in thyroid cells under acute TSH stimulation [31]. Radioiodide accumulation thanks to apigenin-mediated Akt inhibition was also described in PCCl3 rat thyroid cells overexpressing BRAF(V600E) and in primary thyroid tumor cells from TRβ(PV/PV) mice. These results suggest that the outcome of radioiodine therapy for thyroid cancer can be improved by apigenin and other Akt inhibitors given as food supplements [31].

Soy extracts suppressed iodine uptake and increased the protein content of a known autoimmunogenic Tg fragment in Fischer rat thyroid cells (FRTL). These effects might be responsible for the association between higher incidence of Soy consumption with thyroid disorders such as hypothyroidism, goiter, and autoimmune thyroid disease [32].

Among flavonoids, epigallocatechin-3 gallate (EGCG), a catechin abundant in green tea, when administrated to male rats at doses of 25, 50, and 100 mg/kg body weight showed antithyroidal effects as emerged by decreased activity of thyroid peroxidase and 5′-deiodinase I and increased thyroidal Na^+^/K^+^ ATPase activity. In addition, serum T3 and T4 levels were reduced, while serum TSH was elevated in rats, showing in vivo goitrogenic potential [33].

Moreover, the effect of EGCG (10, 40, 60 μM) was also tested on the proliferation and motility of human thyroid papillary (FB-2) and follicular (WRO) carcinoma cell lines. EGCG treatment inhibited thyroid cancer cell growth, reduced cell motility and migration with concomitant loss of epithelial-to-mesenchymal cell transition markers [34]. 

## 5. Melatonin

Melatonin is an indoleamine with different activities in animals and plants, such as anti-aging, antioxidant, circadian rhythm controlling, antiproliferative, or immunomodulatory [35]. 

In a paper published in 1991, both in the Results (“As shown in Table 2, in surviving mice at 19 and 23 months, melatonin treatment resulted in a significant decrease in night levels of T3 and T4 after 7 …”) and in the Discussion (“chronic night treatment with melatonin in the drinking water in aging mice significantly lowers night levels of T3 and T4 in peripheral blood (Table 2) and thus affects aging related thyroid dysfunction by a mechanism yet to be elucidated”), the authors stated that both T3 and T4 decreased after 7 months of melatonin treatment [36]. However, inspection of their Table 2 (see the following Table 2 that was redrawn by S. Benvenga), shows that only the reduction of T3 was statistically significant. Incidentally, another inaccuracy is that such reductions are lower (−20% for T3 and −23% for T4) than those shown in their Table 2 (−25% and −30%).

One note of caution comes from preliminary experiments by the same group in C3H/He female mice that started to be treated with melatonin (10 µg/mL in the drinking water) at 1 year of age. “Melatonin not only failed to prolong the life span of the mice, but, on the contrary, induced a high number of tumors primarily affecting the reproductive tract (lympho- or reticulosarcoma, carcinoma of ovarian origin; histology not shown here) and thus adversely affected the health and survival of melatonin-treated mice” [36]. Indeed, as stated in the Discussion “It was not surprising, in this study, that ovarian tumors developed following chronic melatonin administration, as Kikuchi et al. found that melatonin stimulated in vitro proliferation of a human ovarian KF cell line” [36]. Instead, “a remarkable prolongation of life was seen when NZB mice were chronically given melatonin in the drinking water at night, while no effect was seen when melatonin was given during the day. In spite of the effect of melatonin, the common causes of death in all melatonin-treated or control NZB mice were autoimmune hemolytic anemia, nephrosclerosis and development of systemic or localized type A or B reticulum cell neoplasia” [36]. “A repetition of our experiments by night administration of melatonin in older, aging C57BL/6 male mice resulted again in a significant prolongation of their survival” [36].

At the end of a 4-week duration study in adult male rats, pinealectomy was associated with increased levels of serum FT3 and FT4 levels compared to control rats and, to a greater extent, compared to zinc-deficient rats [37]. The same Turkish team [38] showed that, at the end of a 4-week treatment period with 3 mg/kg/day of zinc and/or melatonin, melatonin has a thyroid function suppressing action, just the opposite to the effect of zinc. However, when zinc is administered along with melatonin, the thyroid function suppression exerted by melatonin is lowered. Just recently, in rats with experimentally-induced thyroid dysfunction, Baltaci et al. [39] found that both melatonin and zinc levels are increased in hyperthyroidism and decreased in hypothyroidism. 

In cultured rat thyroid follicular cells, melatonin increases directly Tg expression, thus regulating TH biosynthetic activity. On the other hand, it has also been reported that thyroid C-cells synthesize melatonin suggesting in the meantime a paracrine role for this molecule in the regulation of thyroid activity [35].

Interestingly, melatonin was found to suppress cell viability, migration and to induce apoptosis in thyroid cancer cell lines in vitro and reduce tumor growth in the subcutaneous mouse model in vivo. In addition, melatonin could enhance sensitivity of thyroid cancer cells to irradiation in vitro and in vivo, suggesting that this molecule may have clinical benefits in thyroid cancer [40]. 

## 6. Omega-3 Polyunsaturated Fatty Acids (Or Fish Oil)

Omega-3 (ω-3) polyunsaturated fatty acids (PUFAs) are docosapentaenoic acid (DPA), α-linolenic acid (ALA), stearidonic acid (SDA), docosahexaenoic acid (DHA), and eicosapentaenoic acid (EPA). Several clinical trials and animal models have suggested that ω-3 possess multiple effects, such as reduction of lipid levels, direct interactions with cytosolic or membrane bound proteins, metabolic effects, alteration of membrane fluidity (after being incorporated into the phospholipid bilayer) or cardiac tissue remodeling and cell-to-cell communications, even if the data demonstrating improvement remain contradictory [41].

Some Authors demonstrated the anti-apoptotic action of ω-3-fatty acids (ω-3 FAs) on cerebellar organogenesis in a murine model of hypothyroidism-induced neuronal apoptosis [42]. Pregnant and lactating rats were first made hypothyroid by methimazole (MMI) administration and then received ω-3 FAs as a mixture of DHA and EPA. Serum levels of T3, T4, TSH, and the cerebellum of postnatal pups at 16 days of age were evaluated. Compared with the euthyroid pups, serum T4 and T3 levels were significantly lower in the untreated hypothyroid and ω-3 FA-treated hypothyroid pups. Thus, ω-3 FA-supplementation caused no significant change in serum T4 and T3 levels in the hypothyroid d16 pups. Compared with the euthyroid and untreated hypothyroid pups, the percentages of EPA and DHA in total cerebellar FAs rose significantly in the ω-3 FA-treated hypothyroid pups. The weight of the cerebellum decreased significantly in untreated hypothyroid pups compared to euthyroids, which was totally recovered upon ω-3 FA treatment of hypothyroids. The cerebellar weight in untreated hypothyroids was about 16% lower than euthyroids and ω-3 FA-treated hypothyroids. The percentage of apoptotic cells in the cerebellum was significantly higher in hypothyroid than in euthyroid pups. However, the apoptotic index of the ω-3 FA-treated hypothyroid pups was not significantly different from that of the euthyroids, but was significantly lower than untreated hypothyroid pups. There was a significantly impaired DNA fragmentation and caspase-3 activation in the developing cerebellum of hypothyroid pups. Upon ω-3 FA treatment the cleaved caspase-3 levels attenuated significantly compared to untreated hypothyroids, nearly reaching the levels of euthyroids. The levels of pro-apoptotic basal cell lymphoma protein-2 (Bcl-2)-associated X protein (Bax) were significantly higher and Bcl-2 and Bcl-extra large (Bcl-xL) were significantly lower in the cerebellum of hypothyroids than in euthyroids. In the cerebellum of ω-3 FA-treated hypothyroids, there was significantly lower expression of Bax and significantly higher expression of Bcl-2 and Bcl-xL compared to untreated hypothyroids. Finally, ω-3 FA-supplementation restored levels of cerebellar phospho (p)-AKT, phospho-extracellular regulated kinase (p-ERK) and phospho-c-Jun N-terminal kinase (p-JNK), all of these molecules being downregulated in hypothyroidism, with no impact on the expression of myelin basic protein, a TH responsive gene. These findings suggest a protective role of ω-3 FAs against cerebellum and brain injury due to fetal hypothyroidism [42].

Another study investigated in adult male rats the effect of hypothyroidism on spatial learning and memory, the underlying mechanisms and the potential therapeutic role of ω-3 supplementation [43]. A subdivision into 3 groups was done starting from 30 male rats: Control, hypothyroid and ω-3 treated. ω-3 FAs supplementation improved memory deficits, increased serum total antioxidant capacity, and also a diminished expression of Cav1.2 protein (the voltage dependent LTCC alpha 1c subunit), together with reduced structural changes, were observed. The data showed that ω-3 FAs could be a useful neuroprotective agent against the cognitive damage that hypothyroidism can induce [43].

TH also have impact on lipid metabolism. For this reason, it has been explored the effect of ω-3 FAs (at dose of 200 mg/kg of body weight/day for 6 weeks) on lipid metabolism among euthyroid, hyperthyroid or hypothyroid Lewis male rats [44]. Hyperthyroid rats had higher fasting blood glucose and plasma postprandial triglycerides levels compared to euthyroid and hypothyroid animals. In contrast, hypothyroid rats had higher levels of total cholesterol, LDL, and HDL cholesterol [44].

A large body of evidence reveals that ω-3 PUFAs have general anti-inflammatory activities and antineoplastic properties. For instance, they act through different mechanisms including alteration of membrane fluidity and cell surface receptor function, modulation of COX activity and increased cellular oxidative stress. The anti-cancer activities exerted by ω-3 PUFAs are also due to their ability to bind the tumor suppressor Peroxisome Proliferator-Activated Receptor gamma (PPARγ) [45,46]. 

Ligand activation of PPARγ induces growth inhibition and apoptosis in different thyroid cell lines, including anaplastic thyroid cancer cells [47,48,49]. Activation of PPARγ could represent a novel treatment option for anaplastic thyroid cancer in order to extend life duration thus warranting a good quality of life [50,51]. 

## 7. Resveratrol

Resveratrol (3,5,4′-trihydroxy-trans-stilbene) is a stilbenoid polyphenol that can be found in various vegetables and fruit, including peanuts, peanut sprouts and grapes. As it seems to have a significant role as either a chemo-preventive and therapeutic agent to treat different diseases [52,53], resveratrol has recently obtained more attention among health professionals and other nutrition experts.

Resveratrol has antioxidant, anti-inflammatory, and antidiabetic effects, in particular its cardiovascular protective actions are associated with various molecular targets, including apoptosis, inflammation, oxidative stress, angiogenesis, mitochondrial dysfunction, and platelet aggregation [53].

In a rat model of subclinical hypothyroidism (SCH), in which SCH is caused by hemi-thyroid electrocauterization, the effect and potential mechanism of resveratrol on memory and spatial learning were studied [54]. The treatment with resveratrol (15 mg/kg) and L-T4 in SCH rats demonstrated an inversion of learning and memory impairment in behavioral test. Resveratrol treatment of SCH rats caused reduced expression of the hypothalamic thyrotropin releasing hormone (TRH) mRNA and decreased plasma TSH. This could indicate that resveratrol treatment would reverse the hypothalamic–pituitary–thyroid (HPT) axis imbalance in SCH rats. Furthermore, resveratrol treatment of SCH rats up-regulated the hippocampal levels of syt-1 and BDNF. In brief, resveratrol treatment improves spatial learning and memory of SHC rats [54].

In another study, by the same team, the possible antidepressant effect of resveratrol was evaluated, after having previously shown that this rat model develops a depression-like behavior [55]. In SCH rats, the over-expression of the hypothalamic TRH mRNA and the high concentration of TSH were decreased to control levels by resveratrol treatment. Compared to SCH rats, resveratrol-treated SCH rats showed a higher preference for sucrose in the sucrose preference test, an increase in breeding frequency and distance in the open field test and a reduced immobility in the forced swimming test. Resveratrol-treated SCH rats had lower plasma corticosterone levels, adrenal gland weight in relation to bodyweight, and expression of the hypothalamic corticotrophin release hormone (CRH) mRNA. In addition to this, resveratrol, on the one hand, adjusted negatively the relative ratio of phosphorylated-β-catenin (p-β-catenin)/β-catenin and expression of GSK3β, and on the other, adjusted positively the relative ratio of phosphorylated-GSK3β (p-GSK3β)/GSK3β and protein levels of p-GSK3β, cyclin D1, and c-myc, in the hippocampus [55]. Altogether, these results indicate that the canonical Wnt pathway was activated in the hippocampus of the untreated model rats and that activation was ameliorated by the resveratrol treatment [36]. The authors concluded that resveratrol exerts anxiolytic- and antidepressant-like effect in SCH rats by downregulating hyperactivity of the HPA axis and regulating both the HPT axis and the Wnt/β-catenin pathway [55].

Fluoride is the most abundant anion in groundwater, creating problems in drinking water and causing metabolic, functional, and structural damage in several organ systems, including structural abnormalities of the thyroid follicles. It was shown that resveratrol supplementation in fluoride-exposed animals prevented metabolic toxicity caused by fluoride, and restored the functional status and the ultra-structural organization of the thyroid [56]. Hence, this study shows therapeutic efficacy of resveratrol as a natural antioxidant in thyroprotection against toxic insult caused by fluoride [37].

The antiproliferative effect of resveratrol depends on the induction of ERK1/2- and p53-dependent antiproliferation in tumoral cells, binding to a specific receptor on plasma membrane integrin αvβ3, and the accumulation of resveratrol-induced nuclear COX-2; in turn, COX-2 combined with ERK1/2, and ultimately with p53, generates a transcriptionally active complex [57]. To date there are conflicting opinions on the preventive and therapeutic abilities of resveratrol. Physiological concentrations of TH (especially T4) interfere with the antiproliferative/anticancer action of resveratrol. This suggests that the in vivo block of the surface receptor for TH on cancer cells, as well as the reduction of circulating levels of T4 and the substitution of T3 (to maintain a condition of euthyroidism), could be used as strategies to recover or potentiate the clinical effectiveness of resveratrol in tumor treatment [57,58].

Resveratrol has been reported to inhibit sodium/iodide symporter (NIS) gene expression and function in FRTL-5 cells, decreasing cellular iodide uptake after 48-h treatment and this effect was also confirmed in in vivo Sprague–Dawley rats [59]. 

Recently, resveratrol has been investigated for its antithyroid effects in vitro and in vivo models. Specifically, in FRTL-5 cells resveratrol has been found to reduce the expression of thyroid-specific genes, such as Tg, TPO, TSHR, NKX2-1, Foxe1, and PAX8 while in rats treated with resveratrol 25 mg/kg body weight intraperitoneally for 60 days a significant increase in thyroid size along with higher serum TSH levels compared with control rats were found [60]. 

Regarding the role of resveratrol as antineoplastic agent, it has been recently reported that this compound inhibits cell proliferation through STAT3 signaling involvement [61] and reverses retinoic acid resistance of anaplastic thyroid cancer cells [62].

More importantly, resveratrol sensitizes selectively thyroid cancer cells to 131-iodine toxicity, while it exhibited radioprotective effects on normal cells, thus for these beneficial actions, resveratrol might improve the treatment of patients with thyroid cancer during radioiodine therapy [63].

In the thyroid setting, the proliferation of thyroid tumoral cells can be stopped by resveratrol, due to the resveratrol-induced increases the quantity and phosphorylation of p53 [1]. Resveratrol also has an action on iodine trapping, for which it appears to be a promising anti-thyroid drug. Overall, the in vitro and in vivo data indicate that resveratrol may act as a thyroid disruptor and a goitrogen, which should be taken into account for potential therapeutic use of resveratrol or as a supplement.

## 8. Selenium

The chemical non-metal element selenium is an essential micronutrient necessary for cellular function. Selenium exerts its nutritional functions in the form of the amino acid selenoCysteine (SeCys) inserted into a group of proteins known as selenoproteins, some of which are the antioxidant enzymes, glutathione peroxidase (GSH-Px) and thioredoxin reductase, and the three deiodinases of thyroid hormones [64]. The major sources of selenium intake are meat and meat products (31%), fish and shellfish (20%), pasta and rice (12%), and bread and breakfast cereals (11%), while the largest selenium concentrations (1 mg/kg) are found in Brazil nuts and offal [64].

One study investigated the improving effects of selenium on cerebrum and cerebellum impairments caused by the MMI-induced hypothyroidism in suckling rats [65]. Pregnant rats were randomized into 4 groups to receive control diet, MMI alone, MMI plus selenium, or selenium alone. Treatments were given from the 14th day of pregnancy until day 14 after delivery. Following the treatment with MMI, a reduction in plasma levels of FT3 and FT4, protein, DNA and RNA contents in cerebrum and cerebellum was observed, in comparison to controls. These parameters improved after cotreatment with selenium. Furthermore, antioxidant enzyme activities (SOD, CAT, GSH-Px) decreased significantly in the group treated with MMI, while malonaldialdehyde (MDA) levels in cerebrum and cerebellum raised. Co-administration of selenium restored these parameters to near normal values. The authors concluded that selenium improved the cerebral and cerebellar damages induced by MMI in suckling rats, and because of such neuroprotection selenium could be used as a dietary supplement against brain impairments [65]. 

Laureano-Melo and colleagues [66] evaluated potential behavioral alterations in offspring of female rats supplemented with sodium selenite during pregnancy and lactation. Selenium supplementation raised T3 and T4 serum levels, decreased tryptophan hydroxylase 2 expression and cholinesterase activity, and increased tyrosine hydroxylase expression in the hippocampus. In childhood, the selenium-supplemented offspring had a decrease in anxiety-like behavior; in adulthood, the locomotor activity and rearing episodes increased in selenium-treated pups. These findings demonstrated that maternal supplementation by sodium selenite induced psychobiological alterations during childhood and adulthood, probably caused by neurochemical changes generated by TH during the critical period of the central nervous system ontogeny [66]. 

One study evaluated the effect of selenium on CD4(+)CD25(+)Foxp3(+) regulatory T cells (Treg) by using an iodine-induced AIT model [67]. This study aimed to explain clinical observations concerning decreased serum levels of thyroid autoantibodies in patients with autoimmune thyroiditis (AIT). NOD.H-2(h4) mice received 0.005% sodium iodine (NaI) water for 8 weeks, and AIT was induced. The group of selenium-treated mice were fed 0.3 mg/L sodium selenite in drinking water. AIT mice showed fewer Treg cells and lower Foxp3 mRNA expression in splenocytes compared to controls (*P* < 0.01). However, both Treg cells and Foxp3 mRNA expression increased after the treatment with selenium, in comparison to untreated AIT mice (*P* < 0.05). Moreover, selenium-treated AIT mice had lower serum Tg antibody (TgAb) titers and reduced lymphocytic infiltration in the thyroid than untreated AIT mice. These findings suggested that selenium supplementation, through the up-regulation of the Foxp3 mRNA expression, can restore normal levels of CD4(+)CD25(+) T cells in mice with AIT [67].

In the thyroid oncology setting, data are available for human cell lines of thyroid malignancy ARO (anaplastic), NPA (BRAF positive papillary), WRO (BRAF negative papillary), and FRO (follicular) cells treated with 150 microM seleno-l-methionine (SM) were assessed for viability at 24, 48, and 72 h. Seleno-methionine treatment was found to inhibit thyroid cancer cell proliferation through the overexpression of GADD (growth arrest and DNA damage inducible) family genes and cell cycle arrest in S and G2/M phases [68].

Although these data are intriguing, the available evidence on the relationship between selenium and thyroid cancer is yet inconclusive [69].

## 9. Vitamins

### 9.1. Vitamin A

Vitamin A deficiency (VAD) and iodine deficiency (ID) are major global public health problems, affecting more than 30% of the population worldwide. VAD can adversely affect thyroid metabolism [70]. A study investigated the effect of concurrent vitamin A and ID on the thyroid-pituitary axis in rats [70]. Weaning rats received for 30 days a diet deficient in vitamin A (VAD group), iodine (ID group), vitamin A and iodine (VAD+ID group), or sufficient in both vitamin A and iodine (control). Serum retinol levels were ~35% lower in the VAD and VAD+ID groups (*P* < 0.001), in comparison to controls and ID groups. No significant differences in TSH, TSH-beta mRNA, thyroid weight, or TH levels, were observed in the VAD and control groups, while they were higher in the VAD+ID and ID groups, and FT4 and TT4 were lower compared to controls. The authors concluded that moderate VAD alone has no measurable effect on the pituitary-thyroid axis, and that concurrent ID and VAD produce more severe primary hypothyroidism than ID alone [70]. Repletion studies in VAD and ID animals suggested: a) In animals with concurrent moderate VAD and ID, primary hypothyroidism does not reduce the effectiveness of high doses of oral Vitamin A; b) VAD does not lower the effectiveness of dietary iodine to correct pituitary-thyroid axis dysfunction due to ID; c) without iodine repletion, high-dose Vitamin A alone in combined VAD and ID could decrease both thyroid hyperstimulation and the risk for goiter [71].

One Chinese study [72] moved from the fact that the interconnections among neural tube defects (NTDs) and TH or vitamin A have been investigated previously but the interaction between the TH and vitamin A pathways were not elucidated. The authors measured the expression levels of TH signaling genes in human fetuses with spinal NTDs associated with maternal hyperthyroidism, and the levels of retinoic acid (RA) signaling genes in mouse fetuses exposed to an overdose of RA on spinal cord tissues [72]. The promoters of cellular retinoic acid-binding protein 1 (CRABP1) and retinoic acid receptor beta (RARB) (both being RA signaling genes) were ectopically occupied by elevated retinoid X receptor gamma (RXRG) and retinoid X receptor beta (RXRB), but had lowered levels of inhibitory histone modifications, indicating that elevated TH signaling improperly induces RA signaling genes. On the contrary, the observed decrease in deiodinase type 3 (Dio3) expression in the mouse model could be explained by raised levels of inhibitory histone modifications in the Dio3 promoter region, indicating that overactive RA signaling could ectopically derepress TH signaling. These data led to hypothesize a potential improper cross-promotion in vivo between two different hormonal signals through their common RXRs, and then histone modifications recruitment [72].

In FRTL-5 cells, all-trans retinoic acid (ATRA) exerts protective role attenuating endoplasmic reticulum (ER) stress-induced alteration of NIS by modulating the phosphorylation of p38 MAPK [73].

ATRA has been also known to induce in vitro radioiodine uptake and to inhibit cell proliferation and invasion of human thyroid carcinoma cells [74,75], thus making this molecule a promising drug able to improve the isotope sensitivity of the most aggressive thyroid carcinoma.

### 9.2. Vitamin D 

Cholecalciferol (or vitamin D3) is synthetized in the skin upon the exposure to ultraviolet B radiation, and it is also introduced from few dietary sources (such as fatty fish). Ergocalciferol (vitamin D2) is synthesized by plants and fungi. Both forms are hydroxylated to 25-hydroxyvitamin D in the liver [76].

Mice, previously sensitized with porcine Tg, and injected intraperitoneally with/without calcitriol (0.1–0.2 μg/kg body weight/die), showed a minor severity of thyroid inflammation vs mice treated with placebo [77]. This effect was even higher in the case of injection with calcitriol and cyclosporine [78].

In another study, mice were pre-treated with intra-peritoneal injection of calcitriol (5 μg/kg every 48 h) before sensitization with porcine Tg. The thyroid did not show the standard inflammation signs compared to controls, indicating a protective role of vitamin D in preventing thyroiditis [79].

The effect of vitamin D was also investigated in animal models of Graves’ disease (GD) [80]. By immunization with adenovirus encoding the A-subunit of thyrotropin receptor, BALB/c mice became model of GD. Hyperthyroid BALB/mice fed with a vitamin D deficient diet showed fewer splenic B cells, decreased interferon-gamma responses to mitogen and lack of memory T-cell responses to A-subunit protein, with respect to mice fed with a regular diet. No differences in TSHR antibody levels were observed. Furthermore, vitamin D deficient BALB/c mice had lower pre-immunization T4 levels and developed persistent hyperthyroidism, indicating that vitamin D is able to modulate thyroid function in this animal model [80].

A study investigated the potential pathophysiological mechanisms for hypocalcaemia in hyperthyroid cats [81]. Hyperthyroid cats had lower ionized calcium levels than healthy geriatric cats, and ionized calcium concentrations were higher in hyperthyroid cats with concomitant or masked chronic kidney disease than non-uremic hyperthyroid cats. Moreover, hyperthyroid cats had higher plasma calcitriol concentrations than control cats. In hyperthyroid cats, hypocalcaemia was not associated with concomitant or masked chronic kidney disease or reduced plasma calcitriol levels. Elevated TH concentrations might influence ionized calcium levels independently from the control by parathyroid hormone and calcitriol [81].

Evidence suggests that vitamin D can negatively regulate the entire process of tumorigenesis, from initiation to metastasis by multiple mechanisms including the regulation of growth factors, cell cycle and signaling pathways [82]. Indeed, it has been largely reported the antineoplastic activities of vitamin D alone and/or in combination with other agents on thyroid cancer cells [83,84,85]. These findings suggest that the activation of vitamin D signaling could be a promising strategy for prevention, as well as treatment of thyroid cancer. 

### 9.3. Vitamin E

Due to its ability to scavenge free radicals, vitamin E is considered an antioxidant. Vitamin E is also very active in the antioxidative protection of thyroid cells membranes, and it is concentrated in the thyroid in control rats, and increased two fold in goiters. Acute and excessive iodine supplementation can cause iodine-induced thyroid cyto-toxicity, that is probably due to an excessive oxidative stress. A study aimed to investigate whether vitamin E could improve iodine-induced thyroid cytotoxicity [86]. Rats received a low-iodine (LI) diet for 12 weeks and developed goiter. A 50-fold vitamin E dose could attenuate two fold iodine-induced thyroid cytotoxicity, even if weight or relative weight of the iodine-induced involuting gland was not diminished by its supplementation, showing that excess iodine can cause thyroid damage and vitamin E can improve in part the iodine-induced thyroid cytotoxicity [86].

In Sprague–Dawley rats, the oxidative stress status of the serum and hippocampus in hypothyroidism, and the effect on cognitive deficit, of L-T4 replacement therapy with vitamin E supplementation, were evaluated. It was shown that L-T4 replacement therapy with vitamin E can improve cognitive deficit in propylthiouracil (PTU)-induced hypothyroidism by decreasing the oxidative stress status [87]. Another study confirmed that L-T4 replacement therapy in combination with vitamin E reduces hippocampus cellular apoptosis index by ameliorating oxidative stress, suggesting that in a hypothyroid rat model the mechanisms of hippocampus tissue damage are associated with hippocampus apoptosis caused by a marked oxidative stress [88].

The role of vitamin E and curcumin has been investigated on hyperthyroidism-induced mitochondrial oxygen consumption and oxidative damage to lipids and proteins of rat liver [89]. Adult male rats received 0.0012% L-T4 in their drinking water and became hyperthyroid, and vitamin E (200 mg/kg body weight) and curcumin (30 mg/kg body weight) for 30 days. Both vitamin E and curcumin have differential regulation on complexes I and II mediated-mitochondrial respiration and were protective against hepatic dysfunction and oxidative stress induced by L-T4 [89].

Another study, conducted in Labeo rohita juveniles fed normal or increased levels of vitamin E and tryptophan for 60 days and then exposed to sub-lethal nitrite for another 45 days without changing their diet, reported that the negative impact on steroidogenesis exercised by environmental nitrites could be bypassed by supplementation of high levels of vitamin E and to a lesser extent of tryptophan [90].

Recently, vitamin E has been found in combination with curcumine and piperine to exert inhibitory effect on cell proliferation through influencing cell cycle regulators such as β-catenin, cyclin D1 and p53 in human thyroid papillary carcinoma cells; however, further studies are necessary to candidate vitamin D as alternative cancer therapy [91]. 

## 10. Zinc

The negative effect of zinc deficiency and positive effect of zinc supplementation on thyroid function of adult male rats (as measured by serum FT3 and FT4 levels) have been mentioned above [36,37]. In these rats, circulating zinc levels are increased in hyperthyroidism and decreased in hypothyroidism [38]. 

In adult male rats, thyroid function has been slightly damaged by the oral administration of 3 mL 30% ethanol [92]. The moderate decrease in serum T3 and T4 and increase in serum TSH was reversed by the 8-week administration of zinc (Zinc sulfate, 227 mL in the drinking water). Of note, serum Zn levels were low upon ethanol feeding, but they were restored to normal levels after Zn supplementation. 

In contrast with the above data on adult male rats [36,37], there are findings from obese mice [93] and from small ruminants [94]. Obese mice and lean controls received a basal diet or a zinc-supplemented diet (200 mg/kg diet) for 8 weeks. After the basal diet, obese mice had lower serum and hepatic T4 and T3 levels than lean mice (*P* < 0.05). Zinc supplementation diminished significantly circulating T4 levels in both groups [93]. A total of 24 healthy male ruminants (12 lambs and 12 goats) were subdivided in 2 groups: Control or Zn group [94]. Control lambs and goats received basal rations alone (40 mg/kg and 35 mg/kg in dry matter, respectively). Both species of animals in the Zn group received a basal ration added with zinc sulphate up to a dose of 250 mg Zn/kg. The treatment lasted for 12 weeks in lambs and 8 weeks in goats. Animals receiving Zn showed more elevated plasma Zn levels than controls during all the experimental period, excluding the 4th week in goats. Compared to controls, the levels of serum total T4 and total T3 were lower in lambs and goats receiving Zn, except in the 4th week. Furthermore, circulating total TH levels of the goats were higher at the 4th week than at the 8th week. Even if a decrease (vs. controls) in the levels of free T4 and free T3 of both small ruminant species in the Zn groups was present, it was not statistically significant [94]. 

In another study, adult male rats were supplemented for 45 days with either zinc (227 mg/L) or magnesium (100 mg/Kg body weight) and then treated with daily intraperitoneal injection of 100 mg/kg body weight of alloxan for 15 days (days 46 to 60) to induce diabetes mellitus. Circulating total cholesterol, triglyceride, and glucose levels were higher while serum T3 and T4 were lower in diabetic rats than controls. Zinc supplementation did not change any parameter in diabetic rats, whereas magnesium decreased the elevated total cholesterol and triglyceride levels of the diabetic rats to the control level [95].

FRTL-5 cell model, derived from a Fischer rat thyroid and displaying follicular cell phenotype, was used to study the effect of zinc depletion, upon the zinc-specific chelator N,N,N0,N0-tetrakis (2-pyridylmethyl) ethylene-diamine, on thyroid function. In this experimental setting which would mimic the in vivo condition, Tg secretion was decreased. Proteomic analyses performed comparing data from zinc depleted/repleted thyroid cells have identified 108 proteins modulated by intracellular zinc status with important physiopathological implications for this endocrine tissue [96].

## 11. Inositol

Inositol is a water-soluble compound strictly related to the vitamin B group (also called vitamin B8). Its most abundant form is myo-inositol [97].

That myo-inositol plays an important role in the thyroid gland can be inferred by the evidence, in male rats, that radioactive myo-inositol is accumulated rapidly (within 1 h) by the thyroid [98]. A previous study in primary cultures of sheep and human thyrocytes demonstrated the TSH regulates myo-inositol transport through an increased phospholipase A2-mediated turnover of phosphatidylinositol and a simultaneous increase in arachidonic acid turnover [99]. Biosynthesis of myo-inositol has been investigated in hypophysectomized and thyroidectomized male rats [100]. It was shown that inositol-1-phosphate synthase is controlled by the pituitary in the reproductive organs and by the thyroid in the liver [100]. 

Myo-inositol is the precursor for the synthesis of phosphoinositides, implicated in the phosphatidylinositol (PtdIns) signal transduction pathway, and it is involved in different cellular processes. In the thyroid cells, PtdIns takes part in the intracellular TSH signaling, via Phosphatidylinositol (3,4,5)-trisphosphate (PtdIns(3,4,5)P3) (PIP-3) [101].

In a recent systematic review on metabolite profile alterations of thyroid cells myo-inositol has been suggested as a thyroid cancer oncometabolite [13].

The effects of inositol supplementation on serum levels of thyroid hormones were evaluated in dairy cows [102]. The supplementation decreased circulating T3 and FT3 concentrations, but not T4 and FT4 concentrations [102].

In humans, it has been shown that the increased levels of TSH declined in patients with AIT and subclinical hypothyroidism, treated with myo-inositol and seleno-methionine. The concentration of both TPOAb and TgAb decreased in both groups. The supplementation with seleno-methionine alone was not able to promote the same reduction [103].

Another paper first showed an immune-modulatory effect of myo-inositol in association with seleno-methionine in patients with euthyroid AIT [104].

A paper reported the beneficial effects of myo-inositol, seleno-methionine or their combination on peripheral blood mononuclear cells (PBMC) exposed in vitro to hydrogen peroxide (H2O2)-induced oxidative stress in both control and women with Hashimoto’s thyroiditis (HT) [105]. PBMC, from 8 HT women and 3 controls, were cultured in the presence of H2O2 alone, or with subsequent addition of myo-inositol, seleno-methionine, or their combination. H2O2 alone decreased PBMC proliferation, and it decreased furtherly and dose-dependently in either group. Moreover, H2O2 alone reduced vitality both in controls and HT women, but vitality was rescued by the three additions, contrasting also genotoxicity. Chemokines levels were increased by H2O2 alone (more in HT women than in controls), and each addition dose-dependently decreased these concentrations in either group, particularly with Myo+SelMet [105].

Another study investigated whether myo-inositol alone, or its combination with seleno-methionine, is effective in protecting thyrocytes from the effects given by cytokines, or H2O2 [106]. H2O2 had a toxic effect in primary thyrocytes increasing the apoptosis, and decreasing the proliferation, slightly reducing cytokines-induced CXCL10 secretion. The interferon(IFN)-γ + tumor necrosis factor alpha(TNF)-α induced secretion of CXCL10 was reduced by myo-inositol+seleno-methionine, in both the presence or absence of H2O2. Seleno-methionine alone had no effect. These findings suggested a protective effect of myo-inositol on thyroid cells [106].

Finally, the beneficial effects of myo-inositol, either alone (2.5 g/kg/day in the drinking water) or administered in association with T3 (30 micrograms.kg-1.day-1 s.c.), were investigated on the cardiac lipid content and function of streptozocin-induced diabetic (STZ-D) rats [107]. The elevations in both plasma and myocardial lipids associated with diabetes were prevented by myo-inositol treatment. Moreover, a partial improvement in cardiac performance of STZ-D rats was observed in the group treated with myo-inositol alone and the group treated with myo-inositol plus T3 [107].

## 12. Conclusions

**Figure 1:**
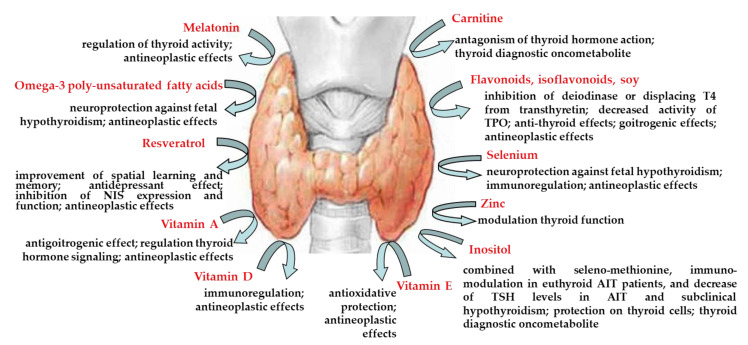
Summary of the main findings. NIS, sodium/iodide symporter; TPO, thyroid peroxidase; AIT, autoimmune thyroiditis.

Nutraceuticals have a place in complementary medicines, defined as a “food, or parts of a food, that provide medical or health benefits, including the prevention and treatment of disease” [2], for the prevention of different pathological conditions, including thyroid diseases. Thyroid supplements have gained lots of attention in the last years. Iodine is the major nutrient for thyroid function, but also other dietary components can have a key role in clinical thyroidology. In this review, we have summarized the cell cultures and animal studies present in literature, focusing on the commonest nutraceuticals generally encountered in the clinical practice (such as carnitine, flavonoids, melatonin, omega-3, resveratrol, selenium, vitamins, zinc, inositol), highlighting conflicting results (Table 3 and Figure 1). These experimental studies are expected to improve the clinicians’ knowledge about the main supplements being used, in order to clarify the potential risks or side effects and support patients in their use.

## Figures and Tables

**Table 1:** Summary of number of articles on given nutraceuticals retrievable on PubMed as of 21 March 2020 *.

n. of Items.	Entry	Humans	Other Animals
1	nutraceuticals	55,737	31,391
2	nutraceuticals AND thyroid	522 (0.9%)	224 (0.9%)
3	carnitine	8134	8778
4	carnitine AND thyroid	71 (0.8%)	95 (1.1%)
5	flavonoids	44,187	49,719
6	flavonoids AND thyroid	222 (0.5%)	248 (0.5%)
7	isoflavonoids	404	281
8	isoflavonoids AND thyroid	4 (0.9%)	4 (1.4%)
9	soy	7965	6531
10	soy AND thyroid	93 (1.2%)	75 (1.1%)
11	melatonin	11,142	14,477
12	melatonin AND thyroid	200 (1.8%)	364 (2.5%)
13	omega-3 polyunsaturated fatty acids	17,168	12,783
14	omega-3 polyunsaturated fatty acids AND thyroid	37 (0.21%)	38 (0.3%)
15	resveratrol	5823	5961
16	resveratrol AND thyroid	54 (0.9%)	42 (0.7%)
17	selenium	13,794	13,888
18	selenium AND thyroid	600 (4.3%)	372 (2.7%)
19	vitamin A	32,637	22,296
20	vitamin A AND thyroid	495 (1.5%)	593 (2.7%)
21	vitamin D	61,418	20311
22	vitamin D AND thyroid	1280 (2.1%)	554 (2.7%)
23	vitamin E	22,004	18,811
24	vitamin E AND thyroid	96 (0.4%)	123 (0.6%)
25	zinc	58,247	50,628
26	zinc AND thyroid	503 (0.86%)	401 (0.7%)
27	inositol	17,144	27,226
28	inositol AND thyroid	147 (0.86%)	205 (0.75%)

* The PubMed search was run using the filter “humans” to exclude “other animals”, and the filter “other animals” in order to exclude “humans”. Note how thyroidal studies account for a tiny fraction of total studies for any listed nutraceutical, and with comparable percentages in humans and animals. For instance, “resveratrol AND thyroid” accounted for 54 of 5823 studies in humans (0.9%) and 42 of 5961 studies in other animals (0.7%).

**Table 2:** Table redrawn from reference #36. In that paper [36], this table was Table 2, and its heading was “Chronic (night) treatment with melatonin modifies night levels of thyroid hormones in serum and maintains the delayed-type hypersensitivity (DTH) response of aging C57BL/6 male mice”.

Groups	Age (Months)	Melatonin (Duration of Treatments, Months)	T3 (ng/mL)	T4 (μg/dL)
Untreated (*n* = 10)	19	------------	0.854 ± 0.165	5.48 ± 1.09
Treated (*n* = 10)	19	3	0.873 ± 0.160 (+ 2.2%) *P* > 0.05 (NS)	5.46 ± 1.51 (− 0.36%) *P* > 0.05 (NS)
Untreated (*n* = 4)	23	------------	0.850 ± 0.028	4.94 ± 1.10
Treated (*n* = 8)	23	7	0.682 ± 0.049 (− 19.8%) * P < 0.001	3.79 ± 1.37 (− 23.3%) § *P* > 0.05 (NS)

* In the original Table, the Authors wrote “(−25%)”. Having noted this error, S. Benvenga wished to repeat statistical analysis with the same test used by the Authors (two-tailed Student’s *t* test). He obtained, *t* = 6.199, which is significant at a *P* < 0.001, confirming the tabulated *P* value. § In the original table, the authors wrote “(−30%)”. Having noted this error, S. Benvenga wished to repeat statistical analysis with the same test used by the Authors (two-tailed Student’s *t* test). He obtained, *t* = 1.450, which is insignificant (*P* > 0.10), thus confirming the tabulated value.

**Table 3:** Summary of the main findings.

Compounds	Main Findings	References
carnitine	antagonism of thyroid hormone action, thyroid diagnostic oncometabolite	[9] [13]
flavonoids, isoflavonoids, soy	inhibition of deiodinase or displacing T4 from transthyretin, decreased activity of thyroid peroxidase anti-thyroid effects goitrogenic effect antineoplastic effects	[16,33] [20,29,30,31] [22] [24,34]
melatonin	regulation of thyroid activity antineoplastic effects	[37,38,39] [40]
omega-3 poly-unsaturated fatty acids	neuroprotection against fetal hypothyroidism antineoplastic effects	[42,43] [45]
resveratrol	improvement of spatial learning and memory antidepressant effect inhibition of sodium/iodide symporter expression and function antineoplastic effects	[54] [55] [59] [57,58,61,62,63]
selenium	neuroprotection against fetal hypothyroidism immunoregulation antineoplastic effects	[65,66] [67] [68]
vitamin A	antigoitrogenic effect regulation thyroid hormone signaling antineoplastic effects	[71] [72] [73,74,75]
vitamin D	immunoregulation antineoplastic effects	[77,78,79,80] [82]
vitamin E	antioxidative protection antineoplastic effects	[86,87,88,89] [91]
zinc	modulation thyroid function	[36,37,38,92,93,94,95,96]
inositol	involvement in the intracellular TSH signaling, via PIP-3 inositol supplementation decreased circulating T3 and FT3 concentrations thyroid diagnostic oncometabolite the treatment, in combination with seleno-methionine, declined the elevated levels of TSH in patients with AIT and subclinical hypothyroidism immune-modulatory effect of myo-inositol in association with seleno-methionine in patients with euthyroid AIT beneficial effects of myo-inositol, seleno-methionine or their combination on PBMC exposed in vitro to H2O2-induced oxidative stress in both control and women with HT protective effect of myo-inositol on thyroid cells myo-inositol, either alone or in association with T3 improved cardiac lipid content and function of streptozocin-induced diabetic rats	[101] [102] [13] [103] [104] [105] [106] [107]

AIT, autoimmune thyroiditis; H2O2, hydrogen peroxide; HT, Hashimoto’s thyroiditis; PIP-3, Phosphatidylinositol (3,4,5)-trisphosphate (PtdIns(3,4,5)P3); PBMC, peripheral blood mononuclear cells.
